# Mesenchymal Stem Cell (MSC)-based therapies for neonatal lung and brain injury – one size fits all?

**DOI:** 10.1186/s40348-026-00262-z

**Published:** 2026-10-07

**Authors:** U. Felderhoff-Müser, J. Herz, I. Bendix, J. Behnke, Y. Dong, H. Ehrhardt

**Affiliations:** 1 Department of Paediatrics I, Neonatology & Experimental Perinatal Neurosciences, University Hospital Essen, University Duisburg-Essen, Hufelandstrasse 55, Essen, 45147 Germany; 2 Center for Translational Neuro- and Behavioural Sciences (C-TNBS), University Hospital Essen, University Duisburg-Essen, Essen, Germany; 3 Department of General Paediatrics and Neonatology, Justus-Liebig- University Giessen and Universities of Giessen and Marburg Lung Center (UGMLC), Member of the German Center for Lung Research (DZL), Giessen, Germany; 4 Department of Neonatology, Charité - Universitätsmedizin Berlin, Berlin, Germany; 5 Cardio-Pulmonary Institute (CPI), German Center for Lung Research (DZL), Universities of Giessen and Marburg Lung Center (UGMLC), Justus-Liebig University Giessen, Giessen, Germany; 6 German Center for Child and Adolescent Health (DZKJ), partner site Berlin, Berlin, Germany

## Abstract

MSC-based therapies have emerged as a highly promising approach for two of the most devastating consequences of perinatal insults such as extreme prematurity and perinatal asphyxia: bronchopulmonary dysplasia (BPD) and neonatal brain injury. Both conditions share a common pathophysiological basis of inflammation, oxidative stress, and disrupted progenitor cell function in critical windows of organ development. Exogenous MSC act primarily through their secretome, releasing extracellular vesicles, growth factors, and immunomodulatory mediators that attenuate injury and support organ development. Despite compelling preclinical evidence and early clinical feasibility and safety data, neither indication has yet achieved definitive proof of therapeutic efficacy in adequately powered randomised trials. This review summarises the current state of knowledge for MSC-based therapies for neonatal chronic lung disease and subsequently for brain injury, highlighting shared mechanisms, organ-specific considerations, and the path towards clinical implementation.

## Introduction

**Fig 1:**
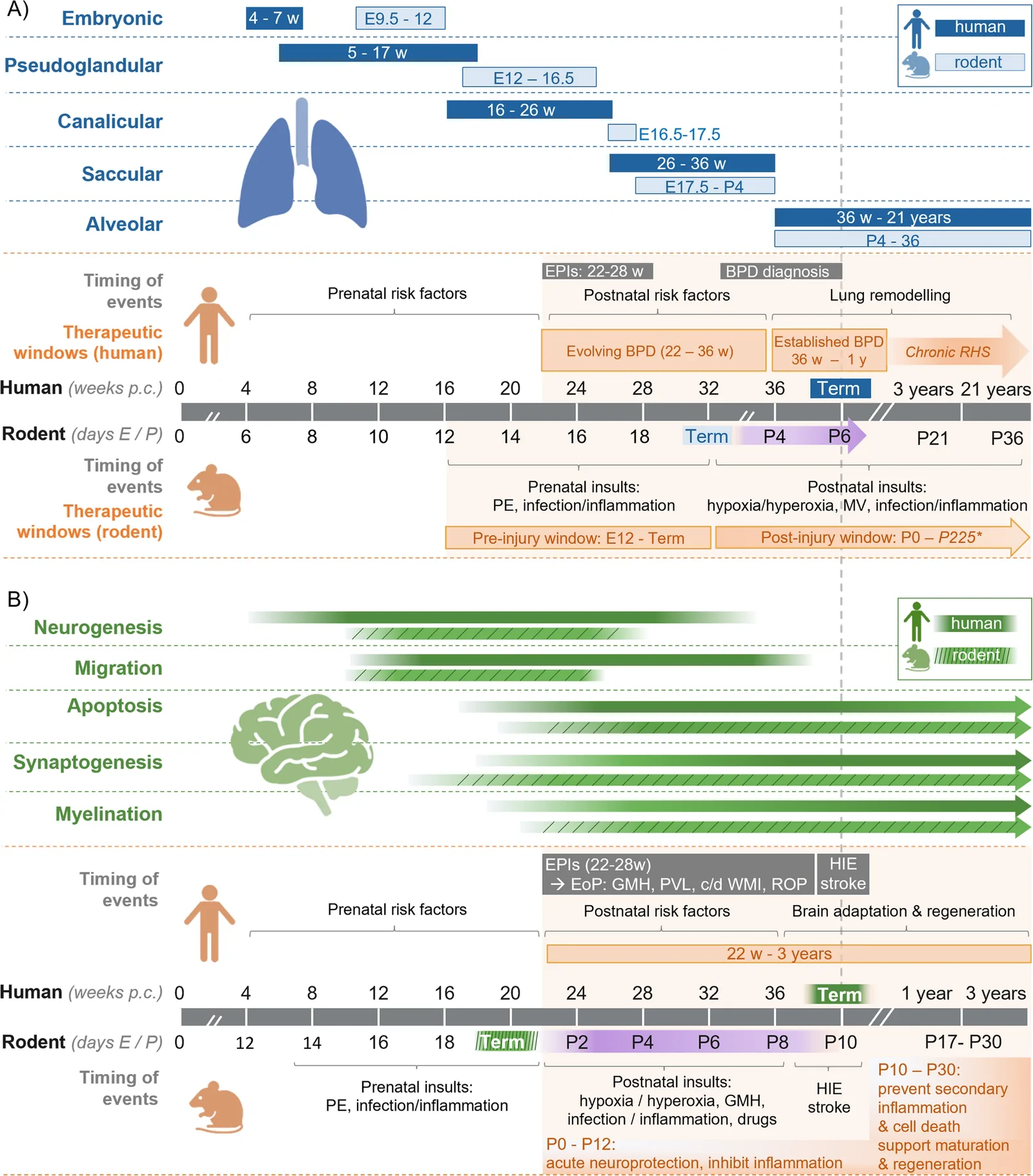
Organ-specific developmental timing, disease-specific vulnerability and windows of therapeutic opportunity for MSC/EV-based interventions in the developing lung and brain. Human and rodent timelines are plotted on a shared axis with species alignment according to organ-specific developmental processes. Rodent organ development is delayed relative to humans, with a larger proportion of the same developmental programme occurring after birth. Processes that take place in utero in humans during the second and third trimester, e.g. late saccular and early alveolar lung development, oligodendrocyte maturation and cortical synaptogenesis, are postnatal events in rats and mice. In (**A**), the rodent scale is linear prenatally and anchored to the human scale at the transition from the saccular to the alveolar stage; in (**B**), the scales are aligned according to cross-species neurodevelopmental processes, placing P10 at human term. The different species alignments reflect that lung and brain reach comparable maturity at different postnatal ages in rodents. Axis breaks spanning the full height of the timeline bar apply to both species and mark the transition to a compressed postnatal scale; partial-height breaks apply to the rodent scale only. Throughout the figure, an arrowhead indicates that the process or window continues beyond the point at which the symbol ends; bars and boxes without an arrowhead are confined to the period shown; a fading bar denotes gradual increases and declines. (**A**) Lung: According to Schittny [1], bars indicate the embryonic, pseudoglandular, canalicular, saccular and alveolar stages of lung development in humans (dark blue) and rodents (light blue), given as weeks post conception (p.c.) and embryonic (E)/postnatal (P) days, respectively. Rodent stage timings refer to mice. In rats, the same stages occur in the same sequence with comparable duration, but at different time points. Extremely preterm infants (EPIs) are delivered during the late canalicular/early saccular stage, i.e. before the onset of alveolarisation. Grey brackets denote the periods of prenatal (pre-eclampsia (PE), infection/inflammation) and postnatal (hypoxia/hyperoxia, mechanical ventilation (MV), infection/inflammation) risk factor exposure and the subsequent phase of lung remodelling. As rodents are born at an earlier stage of lung development, the rodent prenatal period overlaps with the postnatal period of preterm infants. Orange boxes indicate the resulting windows of therapeutic opportunity in humans for prevention during evolving BPD, treatment of established BPD and the potential opportunity to rescue from long-term respiratory health sequelae (RHS), which is based on a recent rodent study [2]. However, confirmative studies are pending. Similarly, in rodents, pre- and post-injury windows relative to pre- and postnatal insults are shown with an enlarged treatment window, recently described for repetitive but not single MSC treatment in 6- to 7.5-month old rats (indicated by an asterisk) [2]. (**B**) Brain: Referring to previous reviews [3–5], shaded bars and arrows indicate the approximate onset, peak and duration of neurogenesis, neuronal migration and apoptosis, synaptogenesis and myelination in humans (solid green) and rodents (hatched green). Rodent bars are offset where the timing of a process differs from humans relative to the aligned scales. EPIs are born during the peak of migration, early synaptogenesis and the pre-myelinating oligodendrocyte stage, which underlies the injury spectrum of encephalopathy of prematurity (EoP: GMH, PVL, cystic/diffuse WMI) and its ophthalmological correlate ROP, whereas HIE, arising from an acute hypoxic-ischaemic insult, and perinatal arterial ischaemic stroke predominantly affect (near-)term infants. Orange boxes indicate the corresponding therapeutic windows: acute neuroprotection and inhibition of inflammation, followed by prevention of secondary inflammation and cell death with support of maturation and regeneration. Similar to evolving BPD, sustained exposures to hyperoxia or infection/inflammation may lead to evolving white matter injury, which would in principle allow a distinction between preventive treatment during exposure and treatment of established injury. However, other perinatal brain injuries such as GMH, stroke or hypoxia-ischaemia are acute and unpredictable events that can occur at any time between 22 weeks and term. For these, therapeutic windows are defined by the timing of the insult rather than by gestational age, comprising an acute/secondary phase amenable to neuroprotection and a tertiary phase amenable to repair and regeneration. The human therapeutic window was therefore not subdivided. Separate rodent windows reflect the typical timing of insults in experimental models (P2–P10). Purple boxes in (**A**) and (**B**) mark the rodent age window in which a single postnatal insult, such as hyperoxia, can elicit the hallmarks of both BPD-like and EoP-like injury in the same animal [6]. Such dual-organ models enable simultaneous assessment of lung and brain outcomes, and therefore of potential organ-spanning effects of MSC- and MSC-EV-based therapies. *Abbreviations*: BPD: bronchopulmonary dysplasia; c/d WMI: cystic/diffuse white matter injury; E: embryonic day; EoP: encephalopathy of prematurity; EPIs: extremely preterm infants; EV: extracellular vesicles; GMH: germinal matrix haemorrhage; HIE: hypoxic-ischaemic encephalopathy; MSC: mesenchymal stem/stromal cells; MV: mechanical ventilation; P: postnatal day; p.c.: post conception; PE: pre-eclampsia; PVL: periventricular leukomalacia; RHS: respiratory health sequelae; ROP: retinopathy of prematurity; w: weeks

Mesenchymal stem cell (MSC)-based therapies have gained considerable potential in regenerative and immunomodulatory medicine in recent years. Their therapeutic potential arises from intrinsic properties including promotion of tissue repair including angiogenesis, modulation of excessive or dysregulated immune responses, and promotion of tissue protection. A major advantage of MSC is the broad immunomodulatory activity and the absence of major short-term safety signals in preclinical and small early-phase clinical studies to date. However, evidence on clinical efficacy largely remains disease- and product-dependent. Although MSC-based therapies represent a promising therapeutic option particularly for conditions characterised by inflammation, immune dysregulation, and impaired tissue repair, evidence in neonatal medicine is limited. However, the field is transitioning from broad experimental enthusiasm towards more standardised, and mechanism-based therapeutic approaches. This review summarises preclinical and clinical evidence on the most devastating clinical conditions in neonatology, Bronchopulmonary dysplasia (BPD), Encephalopathy of Prematurity (EoP) and term hypoxic-ischaemic encephalopathy (HIE), and provides perspectives on future directions. BPD, EoP and HIE differ fundamentally in pathophysiology and temporal evolution, but share common mechanisms of injury approachable with MSC-based therapies. The sections of this review provide a framework with first, the rationale for MSC-based therapies for each of the disease states, followed by a description of the preclinical and clinical evidence. The separate presentation of outcomes following MSC therapy for brain and lung injury derives from the different stages of lung and brain development at birth in rodent models and in preterm infants and the windows of therapeutic opportunity (Fig. 1). Finally, technical aspects including the application of cells or extracellular vesicles as a cell-free option and future challenges are discussed. Details on the therapeutic approach including product identity, source of the cells, autologous vs. allogeneic origin, route of application, timing, dosage and frequency, fresh versus cryopreserved status and application of cells or cell-free options are provided (Table 1). We furthermore discuss clinical trials, the primary and secondary endpoints together with safety outcomes and the availability of follow-up data. For this narrative review, PubMed/MEDLINE and ClinicalTrials.gov were searched for articles and registered trials published until 31 August 2026, using combinations of the keywords “mesenchymal stem cells”, “mesenchymal stromal cells”, “extracellular vesicles”, “secretome”, “cell therapy”, “bronchopulmonary dysplasia”, “neonatal lung injury”, “neonatal brain injury”, “encephalopathy of prematurity”, “white matter injury”, “intraventricular haemorrhage”, “hypoxic-ischaemic encephalopathy”, “perinatal stroke”, “preterm infant” and “newborn”. Original preclinical and clinical studies, systematic reviews and meta-analyses published in English were selected by the authors according to their relevance for mechanisms of action, therapeutic parameters, safety and clinical translation, and reference lists of key articles were screened for additional publications. 

**Table 1:** Comparison of MSC-based therapies for lung (BPD) versus neonatal brain injury (EoP/HIE)

	Neonatal lung injury (BPD)	Neonatal brain injury (EoP/HIE)
Primary MSC source (preclinical)	Bone marrow [7, 8], umbilical cord (blood/tissue) [8–10]	Bone marrow [11, 12], umbilical cord (Wharton’s jelly/cord blood) [13–17]
Primary MSC source (clinical)	Umbilical cord/cord blood [18–20]	Umbilical cord blood or bone marrow [21–24]
Dose range (clinical)	1–2 × 10⁷ cells/kg (intratracheal) [25]	0.5 × 10⁷–5 × 10⁷ cells/kg [21–24]
Dose range (preclinical)	5 × 10⁴–2 × 10⁶ cells/animal [18, 19]	1 × 10⁵–1 × 10⁷ cells/animal [26]
Routes tested (preclinical)	Intratracheal, intravenous [27–29]	Intranasal, intravenous, intraventricular [12–17, 30–32]
Routes tested (clinical)	Intratracheal [18, 19, 33], intravenous [34]	Intravenous [21, 35], intraventricular [22], intranasal [23, 24], (clinical phase I trials only for HIE)
Therapeutic window (experimental)	Most effective prior to/at onset of injury, delayed postnatal application shows reduced efficacy [36–38]	First 24–72 h post-injury in most models [11, 12, 15, 31, 32], intranasal delivery effective up to 10 days post-insult in HIE models [39] and up to 15 days post insult in preterm-birth related brain injury models (systemic inflammation and IVH ) [40]
Standard-of-care interaction (experimental)	Impaired by prolonged hyperoxia/mechanical stretch exposure [41–43]	Variable interaction with therapeutic hypothermia, reduced efficacy in some models, additive benefit in others [12, 13, 15]
Key molecular mechanisms	Attenuation of pro-inflammatory cytokines IL-1β/IL-6/TNF-α/CCL2/CXCL2/TGF-β with preserved anti-inflammatory IL-10 and angiogenic VEGFA/angiopoietin-1 signalling [44–47], pro- ◊ anti-inflammatory macrophage polarisation via decorin/PTX3 [9, 10], reduced myeloperoxidase activity/oxidative stress and microbial dysbiosis [48], sonic hedgehog pathway and preservation of epithelial stem cells [49, 50], stimulation of lung growth factors (KGF, HGF, EGF) and angiogenesis [37, 51, 52]	Attenuation of pro-inflammatory cytokines IL-1β/IL-6/TNF-α with increased anti-inflammatory IL-10 and VEGF [12, 53, 54], neuroserpin normalisation [55], inhibition of microglial/endothelial activation and leukocyte infiltration [12–14], reduced cerebral T-cell infiltration with preserved peripheral T-cell homeostasis [31], pro- ◊ anti-inflammatory microglial/myeloid cell shift [56], promotion of oligodendrocyte proliferation/myelination and neurogenesis [11–14, 57, 58]
MSC-EV evidence (preclinical)	Comparable efficacy to MSC [37, 59], preserves alveolarisation and pulmonary vascular density [60], induce anti-inflammatory macrophage phenotype [61, 62]	Crosses blood-brain barrier after intranasal delivery in HIE and EoP models, reduces neuroinflammation, promotes oligodendrocyte differentiation, repair and neurogenesis [63–67], blocks NLRP3-inflammasome-driven cell death [68], combined with TH improves long-term outcome [69]
Clinical trial phase reached	Phase I–II (largest: *n*=66) [19, 34, 43, 70]	In HIE only, phase I, largest MSC-specific trial in neonatal stroke *n*=10 (PASSIoN) [23, 24], largest overall cell-therapy trial in HIE *n*=23 used cord blood cells [21], Phase II planned for infants with HIE and stroke (i-SToP-CP, *n*=162) [71], no trials for EoP
Clinical efficacy signal	Reduction in severe BPD in some trials, no significant effect on combined primary outcome [19, 20, 34, 70, 72]	Feasibility/safety established, efficacy signals only from small, uncontrolled cohorts [21–24, 35, 73]
MSC-EV evidence (clinical)	One trial (i.v. EV) halted by sponsor before efficacy readout, no safety concerns raised [74]	No clinical trials for both HIE/EoP to date, standardisation of production/potency and scalable manufacturing remain key barriers [75]

*BPD* bronchopulmonary dysplasia, *EoP* encephalopathy of prematurity, *HIE* hypoxic-ischaemic encephalopathy, *MSC* mesenchymal stem/stromal cell, *TH* therapeutic hypothermia

### Aetiology of bronchopulmonary dysplasia

Extreme prematurity (< 28 weeks’ gestation) affects around 1% of all births and BPD constitutes the most severe lung outcome with lifelong restrictions in lung function and pulmonary morbidity [76, 77]. But even in the much larger population of very preterm infants delivered < 32 weeks´ gestation the BPD burden remains high [78]. The aetiology is multifactorial with a long list of pre- and postnatal risk factors disrupting physiological lung development and causing tissue injury to the immature lung [79–81]. The resulting histopathology is characterised by alveolar simplification with reduced surface area for gas exchange, alterations in the composition of the interstitial tissue and rarefication of the pulmonary vasculature [77, 82]. The most important contributor to BPD pathology is the exposure to a hyperoxic environment ex utero and the need to provide supplemental oxygen to safeguard survival of preterm infants. Oxygen-mediated injuries can be separated into direct cell toxicity and induction of pro-inflammatory immune responses in the immature lung with release of pro-inflammatory cytokines and attraction of innate immune cells to the site of injury [79–81]. Further critical determinants of lung pathology are exposures to mechanical ventilation and bacterial infections before and after delivery altering similar signalling pathways as described for hyperoxia. They induce BPD-like changes in animal models on their own or amplify the extent of injury by oxygen toxicity [80, 83].

The simplification of lung structures is a result of rarefication of lung resident mesenchymal stem cells and their phenotype disruption. These cells are critical regulators of further lung development during the saccular and early alveolar stage. Lung resident MSC constitute the most relevant source of lung growth promoting factors and anti-inflammatory cytokines. Furthermore, they are a rich source of proteins of the extracellular matrix and important regulators of cell-to-cell communication and interplay [84–86]. This is why MSC-based therapies attracted particular attention to substitute resident MSC functionality and to equilibrate the physiologic conditions of anti-inflammation and a lung growth promoting milieu as detailed in the subsequent sections [36, 87–89].

### Mechanistic insights into the benefits of cell therapies for the immature lung

In the first study on the application of exogenous MSC, three-day old newborn rats were exposed to hyperoxia and treated with placebo or rat bone marrow-derived MSC which restored lung architecture and attenuated inflammatory cytokine production like TNFα and TGFβ [7]. Further intravenous injection of bone marrow-derived MSC into newborn mice exposed to hyperoxia showed similar benefits. Alveolar simplification and pulmonary vascular remodelling were reduced together with a reduced influx of macrophages and neutrophils into the lungs. Beneficial effects were not executed by the intrapulmonary function of the MSC cells as the therapeutic application of conditioned medium obtained from their cell cultures provided equivalent benefits [27]. Another study investigated the intratracheal application of bone marrow-derived MSC in newborn rats exposed to hyperoxia. MSC treatment resulted in increased survival rates and improved exercise capacity during distance runs and reduced pulmonary arterial acceleration time as correlate of attenuated pulmonary hypertension besides the structural improvements in alveolarization and pulmonary micro vessel density [28]. Lung-protective effects were also observed with conditioned medium of bone marrow derived MSCs, preventing or reverting key features of pulmonary hypertension like peripheral pulmonary artery muscularization [90]. Some pioneering studies also evaluated the beneficial effects of MSC application for the other clinically highly relevant exposure scenario of pre- or postnatal infection. From previous studies and a review of the literature it comes clear, that MSC therapy is beneficial for most deleterious exposures to the immature lung [37, 44, 87, 91].

Investigations executed during the recent years provided unprecedented mechanistic insights into the therapeutic benefit of MSC application. The main efficacy is related to the secretome of the applied MSC which is specified to the extracellular vesicles (EV) of MSC [28, 45, 92]. This is reflected by the fact that MSC application via both, the intratracheal and intravenous route show therapeutic efficacy [27, 28], prevailing large benefits to attenuate inflammatory responses in the immature lung. MSC application attenuated the increase in all studied inflammatory cytokines including the classical candidates like IL-1β, IL6, TNFα, CCL2, CXCL2 and TGFβ. On the other hand, anti-inflammatory properties with cytokines like IL-10 and growth factor signalling including angiopoietin-1 and VEGFA signalling were better preserved [44–47]. Together with these beneficial effects, the recruitment of macrophages to the lung, their conversion into a pro-inflammatory status and the recruitment of neutrophils was largely prohibited. Furthermore, myeloperoxidase activation and oxidative stress were markedly reduced while macrophages were retained with anti-inflammatory properties. Decorin together with the pentraxin-related protein PTX3 were identified as critical drivers of this macrophage polarisation, associated with decreased pro-inflammatory cytokine activity and improved lung structures [9, 10]. These large anti-inflammatory properties were accompanied by less pronounced microbial dysbiosis in the inflamed lung. This aspect is noteworthy as microbial dysbiosis might itself aggravate inflammation and perpetuate or aggravate the vicious circle of lung inflammation and injury [48]. Additional pathways involved in the beneficial effects include the sonic hedgehog pathway and preservation of stem cells of the epithelium [49, 50]. These immunomodulatory and injury repair mechanisms result in overall benefits for the gas exchange and vascular compartment of the lung on a structural and functional basis [27, 93]. Besides the anti-inflammatory properties, the lung growth promoting capacities with the stimulation of keratinocyte, hepatocyte and epidermal growth factors and angiogenesis promoting cytokines are other critical components of MSC action particularly via their secretome [37, 51, 52].

Despite these promising results in rodent models, they could not be recapitulated in the baboon model of preterm birth at the border of viability and subsequent mechanical ventilation with oxygen rich gas for two weeks. While some effects on acute cardiovascular stability were noted, no benefit of the postnatal one-time administration of 10^7^ umbilical cord derived MSC was observed for lung structure and function [94]. These results deserve attention, especially when putting them into the context of a recent publication of in vitro exposure of human umbilical cord derived MSC to hyperoxia and mechanical stretch [41, 42]. Effect sizes were larger for hyperoxia than for cyclic stretch and more pronounced for a high fraction of oxygen [41–43]. For the combined exposure, MSC phenotype changes were permanent during the recovery period after the intervention [41, 42]. These results might explain the lacking benefit of the one-time application in the baboon model with prolonged exposure to cyclic mechanical stretch plus hyperoxia.

### MSC-based therapies to prevent BPD in preterm infants

The promising results from various preclinical trials stands in contrast to the paucity of available data of MSC-based therapies in preterm infants at risk of developing BPD. The first in-human study was performed in South Korea within a phase I trial including 9 extremely preterm infants with an ongoing need for mechanical ventilation beyond the first 5 days of life, using deep frozen umbilical cord blood derived MSCs at a dosage of 1–2 × 10^7^ cells / kg body weight. MSC were administered intratracheally between day 7 and 14 of life. No acute toxicities or side effects were documented. Surprisingly, the intervention group displayed a significant improvement with no severe BPD case and reduction in overall severity stage distribution of BPD. The authors strengthened the results by providing details on the longitudinal course after MSC application that showed non-significant improvements in the respiratory severity score and significantly lower levels of pro-inflammatory cytokines and matrix metalloproteinase-9 in the tracheal aspirates [18]. The follow-up data until the age of two years revealed no safety concerns and a persistent benefit of the MSC therapy with respect to home oxygen supply and hospitalization for acute pulmonary health sequelae [70]. No safety concerns were identified in another phase I study of intravenous umbilical cord-derived MSC administration in very preterm infants with severe BPD [34]. As in the initial study, no infusion-associated adverse events or immediate complications were observed.

The group from the initial study executed a subsequent investigation in a comparable patient population within a phase II trial including overall 66 extremely preterm infants [19]. There were no differences in the combined outcome of death or moderate/severe BPD with 52% in the intervention and 55% in the control group [19]. But again, there were significant improvements in the pro-inflammatory cytokine profile of tracheal aspirates collected after the MSC application. There was only one significant difference detected within the multiple tests executed with a significant reduction of severe BPD cases from 53% in the controls to 19% in the intervention group [19]. In the five-year follow-up study, there was no significant difference in any of the pulmonary outcomes. However, the composite endpoint of any respiratory morbidity including respiratory symptom related hospitalization, emergency department visits or need for oxygen supply was 60.7% in the intervention group while it was 83.9% in the control group. The most pronounced differences on the individual item level were seen for emergency department visits and need for supplemental oxygen [33]. The results from the subsequent study executed on infants of 23- and 24-weeks’ gestation still await publication while patient recruitment was completed (NCT03392467).

An alternative study approach, using amnion cells, resulted in cardiopulmonary compromise following the infusion of the first patient. This severe adverse event recalls the safety concerns from clinical studies in other disease entities that the infusion has to be executed slowly, with the appropriate dilution, stabilizers and filters to avoid vascular embolism [95, 96].

However, the promising results were recapitulated in another study from China including preterm infants < 32 weeks´ gestation [20]. In contrast to the allogenic approach in the previous studies, autologous cord blood mononuclear cells were given intravenously at a dosage of at least 1 × 10^7^ cells / kg body weight with an intended optimal dosage of 5 × 10^7^ cells / kg body weight or placebo within the first 24 h after delivery. 29 infants received the intervention while 33 were infused with placebo. The detailed studies on the blood immune cell composition and cytokine profiles displayed an overall positive effect on T cell numbers and specific T cell subpopulations together with elevated IL10 levels while that of pro-inflammatory cytokines such as TNFα were significantly reduced. The overall incidence of BPD did not differ between the groups. But there were significant differences for moderate/severe BPD with 8 cases in the control group and 1 in the intervention arm [96]. Another study from Australia in infants < 28 weeks used a comparable approach of autologous cord blood mononuclear cells and infusion of 2.5–5 × 10^7^ cells/kg body weight in the second week of life [72]. The authors did not describe any serious adverse events related to the intervention compared to a contemporaneous control cohort. But there was no benefit for the moderate/severe BPD outcome and any of the other endpoints studied [72]. The infants were in the meantime followed-up until the age of 3 months corrected age for their neurodevelopment and no safety concerns were reported [97]. Overall, the association between attenuated local pulmonary or systemic inflammatory response and reduction in the clinically relevant moderate/severe BPD severity stages underlines the therapeutic potential of the approach. The discrepancies in efficacy to reduce severe BPD stages between the trials executed in southeast Asia and Australia raise several questions: Do genetic differences account for the disparity? Are cell preparations of equivalent potency? Are further factors in the clinical management or differences in the crude BPD rate responsible for the disparate results?

Most recently, a phase I study from Canada using deep frozen ready-to-use umbilical cord MSC preparations completed recruitment of 9 extremely preterm infants (HULC-1, NCT04255147). The results on the given safety were presented at various international conferences and were noted in the just published study protocol for the subsequent HULC-2 trial but have not been published officially yet [98]. This subsequent phase II study (NCT07058025) uses repeated intravenous MSC applications and is adequately powered to detect significant differences in the pulmonary outcome. First results are expected to be available by 2028.

Overall, MSC-based therapies remain a fascinating new therapeutic approach to prevent or treat BPD. These perspectives are stipulated by the recent meta-analyses from adult lung diseases like severe COVID-19 infection where in-hospital mortality rates and adverse events were relevantly reduced [99]. This benefit for survival is comparably applicable to adult acute respiratory distress syndrome due to any cause as summarised within another actual meta-analysis [100]. The only phase II trial completed to date was not powered to prove superiority of MSC application for the primary outcome and for the follow-up at 24 and 60 months when limited efficiency is assumed with effect sizes of BPD reduction comparable to that of other therapies [70, 101, 102]. The actual Cochrane meta-analysis does not make any recommendation but demands further research on the topic [103].

### Aetiology of neonatal brain injury

Perinatal brain injury is a devastating condition affecting both preterm and term born infants, leading to significant mortality and lifelong neurodevelopmental disabilities [104, 105].

Preterm brain injury or Encephalopathy of Prematurity (EoP) manifests as germinal matrix/intraventricular haemorrhage (GM-IVH), cystic white matter injury (cWMI) known as periventricular leukomalacia (PVL), clinically seen on serial ultrasound, and diffuse WMI (dWMI), only visible on structural MRI at term equivalent age. The aetiology is multifactorial involving fluctuating cerebral blood flow with impaired autoregulation, systemic inflammation, excitotoxicity from glutamate release, free radical-mediated oxidative injury, and intrinsic vulnerability of oligodendrocyte precursor cells during critical developmental windows [106, 107]. The immature oligodendrocyte lineage, which predominates in white matter during the saccular stage of brain development, is exquisitely sensitive to hypoxia-ischemia, inflammation, and oxidative stress, leading to arrested maturation and subsequent hypomyelination that underlies many long-term neurological sequelae including cerebral palsy, cognitive impairment, and neurodevelopmental disabilities [108]. GM-IVH occurs in 15–20% of very low birth weight infants, with severe grades (III-IV) associated with high rates of cerebral palsy and developmental delay [109].

Rather than resulting from a single, well-defined insult, preterm brain injury arises from the convergence of haemodynamic, inflammatory, excitotoxic, and oxidative insults acting on a developmentally vulnerable substrate, with the relative contribution of each mechanism varying between infants and over time [110]. As a consequence, the therapeutic window during which an intervention could effectively interrupt or modulate the injury process is not clearly defined, and may differ depending on which pathomechanism predominates at a given stage of the evolving injury. This uncertainty complicates both the timing and the design of neuroprotective strategies, including MSC-based approaches, and needs to be considered when interpreting preclinical and clinical evidence.

In term infants, HIE resulting from perinatal asphyxia affects 1–3 per 1,000 live births in developed countries and up to 10–20 per 1,000 in resource-limited settings [111, 112]. HIE initiates a biphasic pattern of injury with an initial phase of primary energy failure characterised by acute cell death, followed by a latent period of apparent recovery, and then a secondary phase occurring 6–48 h after the initial insult featuring mitochondrial dysfunction, excitotoxicity, oxidative stress, inflammation, and predominantly apoptotic cell death [113]. This secondary energy failure phase represents the primary therapeutic target for neuroprotective interventions. Therapeutic hypothermia (TH), initiated within 6 h of birth as the therapeutic time window, has become standard care and reduces death or disability by approximately 25%, yet nearly 50% of infants with moderate-to-severe HIE still experience adverse outcomes [114]. Further critical determinants leading to impaired response to hypothermia are deleterious exposures to inflammation around birth that execute injury via uniform signalling pathway alterations involving microglial activation, cytokine release, and disruption of developmental programs [115].

These ongoing pathomechanisms may also help to explain why the optimal time window for adjunct neuroprotective or regenerative therapies, such as MSC-based approaches, remains less clear than the well-defined 6-hour window for TH. Injury to the developing brain evolves over weeks to months, involving ongoing neuroinflammation, impaired neurogenesis, and disrupted connectivity [107]. A single narrow treatment window may therefore not capture the full period of therapeutic opportunity, and timing may instead need to be tailored to the mechanisms targeted at each developmental stage (Fig. 1). Comparing preterm-birth-related neural maturation deficits with term-born HIE-induced acute cell loss may require different treatment windows, the first supporting ongoing maturation processes in preterms until term born age and beyond, the second inhibiting acute injury and associated inflammatory responses in term-born HIE infants, while supporting tissue regenerative responses in secondary phase. Due to the pleiotropic action of stem cell-based therapies (including anti-apoptotic, anti-inflammatory and pro-regenerative effects), both pre-term and term-related brain injuries can be targeted, though therapeutic windows with regard to insult and cellular target may substantially differ, which needs to be determined in adequate experimental models.

Injury to developing brain structures is a result of both direct neuronal and oligodendrocyte death, reduced proliferation in the neurogenic subventricular and subgranular zone combined with impaired cell maturation [63, 116–119]. Neuronal and oligodendrocyte progenitor cells are particularly vulnerable to noxious stimuli, including hypoxia/ischaemia, hyperoxia and its combination with prenatal inflammation involving microglia activation [118–120]. Zhang et al. highlighted the context-dependent nature of microglia, the brain-resident macrophages, which in a specific cell state can promote oligodendrocyte maturation and myelination, yet selective microglial ablation in certain neonatal models paradoxically exacerbates injury, underscoring that therapeutic modulation, rather than blanket suppression of the microglial response, remains the goal [64]. This is one of the reasons why MSC-based therapies have attracted particular attention to support resident stem cell functionality, and to equilibrate the physiologic conditions of anti-inflammation and a brain maturation promoting milieu as detailed in the subsequent section.

### Mechanistic insights into the benefits of MSC therapies for the immature brain

In a first study by van Velthoven at al. on the application of MSC, 9-day old mice were exposed to unilateral carotid artery ligation followed by hypoxia (Rice-Vannucci model), inducing characteristic features of HIE [11]. Intranasal bone marrow derived MSC administration resulted in a marked reduction of brain tissue loss, the attenuation of pro-inflammatory cytokine production and promotion of oligodendrogenesis and neurogenesis associated with a preferential migration of MSC to the injured brain regions [11]. Importantly, cellular responses translated into improvements in sensorimotor and cognitive function at young adult age [11, 39]. Even delayed intranasal MSC treatment at 10 days post-injury provided long-term effects, making this application route interesting for clinical use [39]. Subsequent investigations expanded the evidence across different injury models and species. Intraventricular umbilical cord blood MSC injection at one hour post HIE in rat pups reduced infarct volume, decreased apoptosis, and improved neurobehavioural outcomes [30]. As in the lungs beneficial effects were not executed by cell replacement as very few MSC engrafted in the brain, yet neuroprotective effects persisted for weeks [121]. This is explained by paracrine mechanisms of MSC, since application of human bone marrow derived MSC-conditioned medium or MSC-derived extracellular vesicles (EV) provided equivalent benefits, suggesting EVs as a major mode of action [122, 123].

To date, more than 100 preclinical studies were published on MSC therapy for neonatal brain injury, executed across multiple species and injury models including HIE, IVH, inflammation-induced dWMI, and neonatal stroke [124, 125]. Shared mechanisms include attenuation of pro-inflammatory cytokines, such as IL-1β, IL-6 and TNF-α, while expression of the anti-inflammatory cytokine IL-10 and of the growth factor VEGF was increased [12, 53, 54]. Neuroserpin was identified as a biomarker whose normalization by MSC therapy correlates with improved neurological outcomes, demonstrating that MSC modulate specific neuroprotective pathways [55]. In addition, MSC’s therapeutic effects were related to inhibition of cellular responses, such as microglia accumulation, endothelial cell activation, and peripheral leukocyte infiltration in rodent models of neonatal HIE [12–14]. Reduced cerebral T cell infiltration but also preservation of peripheral T cell homeostasis was observed in an ovine model of global HIE [31]. Besides modulation of adaptive immune cells, a shift in microglia cell polarisation from a pro-inflammatory cell state secreting TNF-α and IL-1β to an anti-inflammatory phenotype with increased TGF-β and IL-10 expression was noted in a preterm WMI injury model following human umbilical cord MSC-treatment [56]. Several pathways critically involved in maturation were regulated towards physiologic conditions with exogenous MSC application. These include increased oligodendrocyte proliferation and myelination [11–14], enhancement of neurogenesis [11, 13, 57], all together resulting in benefits for the development and recovery of brain structure and function [11–14, 39].

In the clinical setting of HIE, combination with TH is mandatory, with multiple studies demonstrating additive effects as reviewed by Mintoft et al., who also presented preclinical evidence from a piglet HIE model that intranasal umbilical cord-derived MSC augment the neuroprotective effects of TH [126]. However, not all preclinical evidence supports a straightforward benefit of combining MSC with TH, since combination with delayed intranasal mouse bone marrow MSC application in a neonatal mouse HIE model resulted in reduced therapeutic effects on cognitive functions and myelination. The altered brain microenvironment 3 days following TH modulated MSC function. Co-culture experiments revealed increased pro-inflammatory cytokine and decreased growth factor expression upon exposure to brain extracts from hypothermia-treated animals [12]. However, in piglets, intranasal administration of human umbilical cord MSC earlier and repeatedly was shown to provide additive protection to hypothermia [15]. In a comparison to bone marrow MSC, umbilical cord-derived MSC provided benefit on grey matter tissue loss compared to TH monotherapy, even when administered three days after TH [13].

Taken together, the attention to MSC-based therapies is justified for the multiple beneficial actions, the large effect sizes with reductions in injury volume of 30–50%, and benefits for various structures such as grey and white matter, and neurovascular function. However, rodent and large animal models represent idealized research scenarios with a defined injury and the possibility for long-term follow-up studies, while in the clinical setting the immature brain may be exposed to multiple and repetitive perinatal insults. Therefore, preclinical models combining insults have been established [119, 127, 128].

### MSC-based therapies to prevent or treat neonatal brain injury in human neonates

Despite obvious preclinical evidence, the large number of clinical studies in BPD is in contrast to studies available on brain injury. The first in-human study for HIE was performed by Cotten et al. as Phase I trial in 23 term infants with moderate-to-severe HIE receiving TH. Autologous umbilical cord blood cells (not MSC) were administered intravenously (i.v.) between 1 and 14 days of life. The study demonstrated feasibility of cell collection, processing, and administration with no serious adverse events. One-year neurodevelopmental outcomes showed 77% favourable outcomes, though the small sample size and lack of controls precluded definitive efficacy conclusions [21].

A Phase I trial of allogeneic umbilical cord blood-derived MSC in 6 infants with HIE and TH, used one or two doses of 2 × 10^6^cells/kg administered i.v. Treatment was well-tolerated treatment across all dose levels. Developmental assessment by standard scores obtained between 12 and 17 months were within the average to low-average range for age [35].

A first Phase I dose-escalation trial in extremely preterm infants (mean gestational age 26.1 weeks, mean birthweight 808 g) with IVH (Grade III, IV) received intraventricular injection of allogeneic umbilical cord blood-derived MSC (5 × 10⁶ cells/kg, *n* = 3, 1 × 10⁷ cells/kg, *n* = 6; at 11.6 postnatal days). Treatment was well-tolerated and long-term follow-up data showed encouraging neurodevelopment though controlled efficacy trials are needed [22].

Another Phase I study (PASSIoN) included 10 infants with perinatal arterial ischemic stroke (PAIS), receiving 5 × 10^7^ bone marrow–derived MSC intranasally within 7 days of presentation [23, 24]. After confirming feasibility and safety, MRI at 3 months showed less asymmetries of the posterior limb of the internal capsule, and two-year follow up revealed a better motor performance compared to a historic registry cohort [23, 24]. Based on this a Phase II trial (i-SToP-CP) is planned for treatment of 162 HIE and PAIS patients with intranasal delivery of bone marrow MSC [71]. A 2025 systematic review and meta-analysis investigating pooled outcomes from four studies comprising 153 neonates revealed trends towards improved neurodevelopment with no safety concerns, though statistical power was limited by small sample sizes [73].

MSC-based therapies remain a feasible therapeutic approach for neonatal brain injury involving extensive analysis of stem cell fate in HIE [129–131]. The association between reduced microglia and astrocyte reactivity with enhanced IL-10 levels in the blood and balanced brain metabolites assessed via brain imaging in newborn lambs underlines the therapeutic potential of cell therapies [132, 133]. However, adequately powered clinical trials are mandatory to establish MSC-based therapy adjunctive to TH for HIE and expanding indications for other forms of brain injury. Critical issues regarding dosage, timing, delivery route, heterogeneity and cellular plasticity in a complex tissue environment remain to be resolved.

### Technical aspects and challenges of MSC therapy for both aetiologies

Beyond the source and choice of cell product, the technical implementation of MSC-based therapy critically determines therapeutic success. A key underlying principle is homing, the directed migration of administered MSC towards sites of injury, guided by chemokine and adhesion molecule signalling such as SDF-1/CXCR4. Following i.v. administration, however, a large proportion of MSC become trapped in the pulmonary microvasculature, which favours passive targeting of the lung but limits the fraction of cells reaching the brain. This route-dependent biodistribution has shaped the choice of organ-specific delivery strategies discussed in this chapter, including intratracheal application for the lung and intranasal or intraventricular application for the brain. As therapeutic efficacy does not require sustained engraftment but is largely mediated by transient paracrine signalling, source, dosing, route of application, timing and cell-free MSC-EV approaches as additional determinants of therapeutic outcome will be addressed.

In neonatal lung and brain pathologies, preclinical studies were executed mostly with MSC obtained from the umbilical cord or bone marrow. However, umbilical cord blood, Wharton’s jelly, placenta and adipose tissue besides the amniotic fluid are further sources of MSC [37, 134–136]. The direct comparison of umbilical cord and bone marrow derived MSC at equivalent dosages showed lung protective effects when applied 3 days after start of hyperoxia exposure, but MSC from the umbilical cord displayed superiority with respect to the suppression of inflammatory macrophage influx and morphometric parameters of lung alveolarization [8]. A similar comparison in the same study is lacking for brain injuries. However, comparing outcomes in the setting of HIE and TH, the use of more immature umbilical cord -derived MSC seems to be superior to adult bone marrow derived MSC [12, 13, 15, 32, 126]. The umbilical cord has unique advantages, given its availability at birth, immune-privileged properties and superior proliferative capacity compared to adult bone marrow MSC [137, 138]. A recent systematic review on animal models of inflammation with freshly cultured versus cryopreserved, ready to use from the shelf products concluded that efficacy is mostly comparable arguing for using cryopreserved standardised high-quality products [139]. This refers to the immunomodulatory potency and as well further MSC properties like antimicrobial activity [140].

Dosing seems to be critical in both injury types. In the lung, therapeutic effects were observed for 5 × 10^4^ up to 2 × 10^6^ cells applied per animal with a well-described dose-effect relation [25]. For neonatal brain injuries, therapeutic effects were observed for doses ranging from 1 × 10⁵ to 1 × 10⁷ cells per animal in rodent studies [26]. In human Phase I clinical trials, 0.5 × 10⁷ to 5 × 10⁷ cells/kg were applied, however in different pathologies (HIE, IVH, stroke) with different delivery modes [21–24].

Timing of delivery might outweigh dose if administration is within the critical “window of opportunity.” For both pathologies, concerns about efficacy remain with the time-delayed application of MSC. However, this is the most realistic option for the clinic, although efficacy may be lower compared to application prior or with start of exposure as demonstrated in the BPD setting [37, 38, 87]. Similarly, in small and large animal models of neonatal brain injury therapeutic efficacy was demonstrated for application during the first 24–72 h post-injury [11, 12, 15, 31, 32]. Lung-protective effects were observed in the rodent model when MSC were applied at 3 months after the end of exposure to hyperoxia or when repetitively applied between 6 and 8 months of life [2]. In line, beneficial effects were observed in a neonatal HIE model with intranasal application at 10 days after the insult [39]. These results raise the hope that lung and brain regeneration is in principle feasible as for example observed for lung growth promoting factor FGF10 [2, 141] and stimulation of endogenous repair including neurogenesis and remyelination following MSC therapy in brain injury [11, 142].

Besides standardization of manufacturing processes, systematic dose-finding studies represent critical priorities for advancing clinical translation [143]. These aspects have been recently addressed in a European Research Consortium (EU Horizon, PREMSTEM, https://www.premstem.eu/). Using the same MSC batch across different sites, therapeutic capacity was investigated in various small and large preclinical models of EoP including perinatal noxious stimuli. One of the first published studies of the consortium compared three different doses, three different time points of delivery and intravenous vs. intranasal delivery in an inflammation-driven EoP model, resulting in 18 treatment groups. Applying a computational biology approach, it was shown that intranasal delivery of 20 × 10^6^/kg at postnatal day 20 revealed the largest efficacy with regard to microglia modulation and myelination in a germinal matrix haemorrhage model [40]. In a rodent model of neonatal HIE, kinetic analyses revealed efficacy of intranasal therapy, when applied up to 10 days after the insult but not later [39]. A second recently published in vitro study in a hyperoxia model of EoP indicated that indirect hMSC co-culture mitigates hyperoxia-induced impairment of immature oligodendrocytes and hippocampal neurons and that hypoxic preconditioning may modulate this effect in a cell type-specific manner [58].

The optimal delivery route of MSC should balance the potential benefit against the administration route particularly in critically ill neonates. To date, it is not possible to conclude which route is more effective to prevent BPD as it is not feasible to secure the equivalency of the applied dosages [29]. Studies concluded that homing to the injured lung is one additional feature of systemic MSC therapy besides the large effect size by the MSC secretome [144]. For the brain intranasal, intravenous, and intraventricular routes showed therapeutic efficacy [12–17, 30–32]. Importantly, studies in small and large animals models demonstrated large benefits to attenuate neuroinflammation via the less invasive routes of intranasal or systemic administration [12–15, 17, 31, 39]. Intranasal delivery exploits olfactory and trigeminal pathways for direct nose-to-brain transport, with MSC detected in parenchyma within hours and accumulation in injured regions [39]. A key advance in understanding intranasal delivery was the identification of CXCL10 as a critical chemoattractant enabling efficient migration of MSC to the site of injury [145]. Intravenous administration is clinically feasible, though first-pass lung trapping reduces brain delivery efficiency. Nonetheless, systemic anti-inflammatory effects and secretion of diffusible paracrine factors still provide neuroprotection. Intraventricular administration has been tested clinically for severe IVH which demonstrated safety and potential efficacy [22]. However, due to the invasive procedure safety concerns should be considered.

MSC heterogeneity and senescence remain key bottlenecks limiting clinical translation and large-scale manufacturing of cell-based therapies. Although cellular heterogeneity of umbilical cord derived MSC was limited, concerns remain in light of the heterogeneity and functional vulnerability as described for lung resident MSC. This becomes particularly important when larger cell numbers are required [75, 146, 147]. Besides heterogeneity of MSC between different tissue sources, inter-donor variability, heterogeneity of cell clusters derived from the same tissue source and changing phenotypes during cell culture pose challenges to generate an off-the-shelf product [43, 147–149]. It has been recently reviewed how the quality of the product and function of their secretome might influence the product’s efficacy [150].

A future research task will be to evaluate how sex of donor and recipient interact. From a first study in neonatal hyperoxia-induced lung injury it can be assumed that female sex has superior properties in male recipients [151]. Furthermore, aged MSC have reduced immunomodulatory capacity [152], and the immaturity of cells from preterm donors itself may provide a further advantage [153, 154]. However, the endotype of preterm birth itself may impact the MSC phenotype in the umbilical cord with enrichment in genes representative of fibroblasts and markers of cellular senescence which equates to loss of functionality [41, 148, 149]. Surprisingly, phenotype alterations were also observed in cells derived from preterm infants delivered for preeclampsia [148]. Variable effect sizes and lung outcomes in the rodent LPS-BPD model depending on the onset and strength of amniotic infection argue for a careful consideration of the endotype of prematurity to select the best donors for the cell-based therapies [155]. A solution to overcome limitations associated with MSC heterogeneity is presented by immortalization of MSC, to select for the best cell line with most protective capacities based on in vitro characterisation and in vivo models [63, 75]. The use of immortalized clonal MSC may not fully eliminate variability, as factors such as developmental processes, epigenetic modifications, or genetic drift could lead to the re-emergence of heterogeneity or tumorigenesis [75, 156].

Improving the therapeutic efficacy of MSC attracted attention during the recent years. Hypoxic preconditioning (1% O_2_ for 24 h before use) demonstrated advantages with respect to alveolar and microvessel development and beneficial effects on pulmonary hypertension [157]. It also enhanced the therapeutic efficacy of intranasally administered MSC in a neonatal HIE mouse model and in an in vitro model of hyperoxia-induced EoP, as described above [58, 158]. These MSC outperformed normoxic MSC in reducing lesion size and sensorimotor impairments, and showed superior migratory capacity, enhanced neural stem cell differentiation, and exhibited elevated abundances of extracellular matrix remodelling proteins [158]. Additional approaches intend to improve the efficacy of MSC by genetic modification. Genetic inhibition of CCL2 proved highly efficient to reduce pro-inflammatory cytokines, M1 polarisation of macrophages and support of lung alveolarization [159]. In neonatal HIE, intranasal therapy with MSC overexpressing BDNF resulted in improved motor function and decreased lesion volume compared to unmodified MSC [160]. In a neonatal stroke model, MSC- and BNDF-hypersecreting MSC were equally effective [57].

Concerns with respect to MSC’s safety profile extend beyond the lung-specific phenotype alterations discussed above and include immunogenic potential, theoretical tumorigenicity, and thrombogenic or procoagulant effects following intravascular administration, as well as broader manufacturing- and quality-control-dependent risks arising from donor variability, culture-induced phenotypic drift, and the lack of standardised potency assays. Notably, the absence of major short-term safety signals across the clinical trials discussed in this review should not be equated with established long-term safety, particularly regarding repeated dosing and developmental outcomes, which remain to be systematically assessed. These aspects have been comprehensively reviewed elsewhere [161] and are only briefly summarised here. Immunocompromised preterm infants do not have a well-developed allogenic defence control and MSC themselves are capable of adapting their immunologic properties. This may result in evasion of immunologic control, raising fears about potential tumorigenicity [156, 162, 163]. On the other hand, MSC may change their immunomodulatory capacity in in vitro culture for cell expansion and can thereby make themselves immunogenic and become prone to elimination. Future projects should address this point and need to strictly control any detrimental exposure [163]. In vivo, lung resident MSC can change their phenotype into an inflammatory or myofibroblastic phenotype when exposed to the adverse stimuli [85, 86, 147]. Similar constraints apply to exogenously applied MSC, showing phenotype alterations depending on pre- or postnatal exposures [41, 149]. In support of this, MSC change their phenotype in response to an altered brain tissue environment following TH [12].

Taken together, the optimal MSC source and preparation, the minimum effective dose, and the choice of administration route to maximise organ delivery and therapeutic efficacy remains to be determined [150]. Further priorities include cell manufacturing and characterisation, validated potency assays predictive of clinical efficacy, optimal timing, selection of clinically meaningful endpoints including long-term follow-up. The need for repeated dosing, the identification of biomarkers for patient selection and response monitoring are likewise important. Even when these requirements are met, the central challenge remains the cellular plasticity of MSC in response to the lung and brain microenvironments. The combination of low MSC engraftment in both target organs with substantial, long-lasting therapeutic benefit [28, 39], points to paracrine-mediated protection via EV release.

### Extracellular vesicles – a cell-free option to prevent BPD and neonatal brain injuries

The transition from whole-cell MSC preparations toward EV-based approaches may offer some advantages in safety, scalability, and compatibility with neonatal intensive care interventions, e.g. TH. Tumorigenesis and cellular plasticity dependent on the tissue environment could also be overcome.

In BPD, remarkable evolution from whole-cell to EV-based approaches is well documented. A recent study showing that bone marrow MSC-derived conditioned medium prevented CPAP-induced airway hyperreactivity in newborn mice, preserving physiological airway function [164, 165]. Human and rodent studies on MSC-EV application, univocally demonstrate comparable efficacy [37, 59]. In addition to effects of exogenous EVs, endogenously released EVs are critical determinants of lung development with changing properties over the different developmental stages [166]. For exogenous MSC-EV application, it was shown in a rat model of hyperoxia-induced lung injury that MSC-EV preserved alveolar development, induced transcripts related to vascular development and improved pulmonary vascular density that persisted into adulthood. The route of administration via intratracheal or intravenous application displayed equivalent efficacy but EV from Whartons Jelly MSC had superior properties compared to bone marrow MSC [60]. In newborn BPD mice, MSC-EV co-localised and restored the fraction of alveolar macrophages during hyperoxia and induced an immunosuppressive myeloid cell phenotype with epigenetic and phenotypic reprogramming [61]. In newborn rats exposed to hyperoxia the beneficial effects of EV on lung morphometry, associated with preferential anti-inflammatory macrophage polarisation were confirmed [62]. In a mouse preeclampsia model of lung injury, weekly administration to pregnant mothers results in preservation of lung growth promoting factors, reduced intra-amniotic inflammation and improved lung development [167]. Therapeutic application of MSC-EV in chorioamnionitis reduced intrauterine inflammation and preserved alveolar development, lung mechanics, pulmonary vessel density and reduced right ventricular hypertrophy [168]. Intratracheal EV application after intraperitoneal injection of LPS and before start of the exposure to mechanical ventilation resulted in preserved neural progenitor cell function arguing for a systemic effect or a tight connection between lung and brain [169]. First experimental data point towards a lung-brain axis, with communication pathways involving cytokines but also extracellular vesicles [170, 171].

In models of neonatal brain injury, release of EV-containing mRNAs, microRNAs and further subcellular fractions came into focus of research within the past years [63–67, 69, 116, 123, 172, 173]. Focussing on preterm white matter injury, intranasally administered MSC-EVs cross the blood-brain barrier, accumulate at injury sites, reduce neuroinflammation, promote oligodendrocyte differentiation, and improve behavioural outcomes in preclinical models [64]. Independent of injury type and developmental stage, reduced microglia activation, decreased pro-inflammatory cytokine expression, improved myelination, increased neurogenesis and BBB integrity at subacute disease phases were described all together translating into improved long-term motor-cognitive outcome [63–67, 69]. At the molecular level EV from human umbilical cord MSC protect from neonatal brain injury by blocking NLRP3-inflammasome-driven cell death [68]. Labusek et al. demonstrated that EV from immortalized mesenchymal stromal cells protect against neonatal HIE, offering potential advantages for scalable manufacturing and reduction of heterogeneity of donor cells [63]. Importantly, their combination with TH but not TH monotherapy, improved long-term cognitive and neuropsychological deficits [69]. Furthermore, modification of the MSC secretome through specific preconditioning protocols can prolong the regenerative treatment window for neonatal brain injury addressing practical limitations of delayed treatment in clinical settings [174].

MSC-EV application in clinical trials suggest efficacy may be optimal within a relatively narrow dose range, highlighting importance of careful dose optimisation [175]. Unfortunately, the only study (NCT03857841, UNEX-42) to determine the safety and efficacy of EV in preterm infants at high risk for bronchopulmonary dysplasia also including brain imaging as secondary endpoint was halted by the sponsor after onset of recruitment. The decision to stop recruitment was not based on concerns of safety or efficacy [74]. In support of this across other acute and chronic diseases, the limited available data argue for the safety and potential efficacy of MSC-EV [176]. However, no specific trials targeting brain injury exist, possibly due to the large-scale manufacturing issues. The Second Neonatal Cell Therapies Symposium emphasised the need for standardised outcome measures, industry partnerships, and meaningful stakeholder engagement including families affected by neonatal brain injury [54].

Similar unresolved questions as for MSC remain for MSC-EV. There is evidence from amniotic cells as source of the secretome that EV from immature donors might be superior compared to more mature donors. It has not been well established whether there are differences in EV properties between the various sources [7, 154, 177, 178]. For safe and efficient introduction into clinical care it is mandatory to standardise EV production, purification, and storage conditions and to assess functionality and therapeutic potency, since the lack of standardised protocols for MSC culture and EV isolation currently leads to substantial batch-to-batch variability in EV yield, cargo composition, and potency, and no harmonised assays yet exist to define EV identity and dose [75].

### Summary: MSC-based therapies for neonatal lung and brain injury

The current evidence for MSC-based therapies in neonatal lung and brain injury reveals a parallel trajectory across both organ systems, underpinned by shared biology but shaped by organ-specific constraints and developmental windows (Fig. 1; Table 1). Nevertheless, both conditions are characterised by inflammatory dysregulation, disrupted progenitor cell function, and arrested development during critical windows of organ maturation, making them receptive to the same core MSC targets. To address this pre-clinically, the development of animal models covering both pathologies, thereby allowing assessment of MSC-based therapies on both organs simultaneously, is needed [6, 171]. In support of this, a recent study showed that intratracheal administration of MSC simultaneously attenuated impaired lung development and hippocampal cell degeneration [179]. In both fields, mechanistic understanding has converged on the secretome as the primary mediator of action. The preclinical evidence base is substantial with consistently demonstrated structural and functional benefits across multiple injury models. It will be important to specify the optimal therapeutic window for the different diseases and to evaluate MSC-based therapies in the context of standard treatment.

Clinical evidence remains limited to small phase I and II trials. To date, no major short-term safety signals have been identified, although these trials were neither designed nor powered to establish in particular long-term safety. For BPD, the Korean phase I and phase II trials did not identify major short-term safety signals following intratracheal MSC administration and showed reduced airway inflammation, with five-year follow-up data suggesting a lower overall respiratory morbidity, while the primary outcome of death or moderate/severe BPD was not improved [18, 19, 33]. For neonatal brain injury, the PASSIoN trial in ten infants showed that intranasal MSC delivery in perinatal arterial ischaemic stroke is feasible without major short-term safety concerns, with encouraging two-year neurodevelopmental outcomes compared to a historical cohort [23, 24]. Both fields share unresolved challenges: optimal cell source, donor age, dose-response relationships, timing, and the need for repetitive dosing. A shared concern is the interaction with standard intensive care interventions since data from the lung show that prolonged hyperoxia and mechanical ventilation impair MSC viability. Data from the brain suggest that combining whole MSC with TH can reduce the cells’ therapeutic capacity, a problem possibly overcome by switching to MSC-derived EV. Clinical trials are underway in both indications and their results will determine whether MSC-based therapy may become standard adjunctive care for critically ill neonates in whom protecting lungs and brain simultaneously is the ultimate goal. It is still too early to draw conclusions on the practicability of a one-size-fits-all concept. The available evidence supports shared therapeutic principles across BPD, EoP and HIE, namely secretome-mediated immunomodulation, attenuation of inflammation and oxidative stress, and support of progenitor cell function and organ maturation, but not a uniform MSC product, route of administration, dose or treatment window. These parameters need to be defined separately for each disease entity and developmental stage, and in the context of the respective standard of care such as TH in HIE. Priority must be given to long-term clinically relevant health outcomes, even where this slows short-term progress in the field.

The shift towards MSC-EV as a cell-free approach has gained momentum in both fields. Intratracheal EV preserved not only pulmonary architecture but also neural progenitor cell function, pointing to shared inflammatory signalling or systemic EV trafficking as a potential avenue for simultaneous lung and brain protection [169]. Currently, many major unresolved issues remain regarding whether EVs are a therapeutic alternative, including product definition, isolation, dose metrics, cargo heterogeneity, storage, potency testing, biodistribution, and scalable manufacturing. Moreover, most importantly robust neonatal clinical evidence for MSC-based therapies is mandatory, yet currently lacking.

## Data Availability

No datasets were generated or analysed during the current study.
